# Medical-Grade Silicone Rubber–Hydrogel-Composites for Modiolar Hugging Cochlear Implants

**DOI:** 10.3390/polym14091766

**Published:** 2022-04-26

**Authors:** Suheda Yilmaz-Bayraktar, Katharina Foremny, Michaela Kreienmeyer, Athanasia Warnecke, Theodor Doll

**Affiliations:** 1 Department of Otolaryngology, Hannover Medical School, Carl-Neuberg-Straße 1, 30625 Hannover, Germany; foremny.katharina@mh-hannover.de (K.F.); kreienmeyer.michaela@mh-hannover.de (M.K.); warnecke.athanasia@mh-hannover.de (A.W.); doll.theodor@mh-hannover.de (T.D.); 2 Cluster of Excellence Hearing4All, Carl-Neuberg-Straße 1, 30625 Hannover, Germany; 3 Fraunhofer Institute for Toxicology and Experimental Medicine (ITEM), Nikolai-Fuchs-Straße 1, 30625 Hannover, Germany

**Keywords:** sensorineural hearing loss, cochlear implants, self-bending electrode arrays, silicone rubber–hydrogel composites, actuators, swelling behavior, curvature, biocompatibility

## Abstract

The gold standard for the partial restoration of sensorineural hearing loss is cochlear implant surgery, which restores patients’ speech comprehension. The remaining limitations, e.g., music perception, are partly due to a gap between cochlear implant electrodes and the auditory nerve cells in the modiolus of the inner ear. Reducing this gap will most likely lead to improved cochlear implant performance. To achieve this, a bending or curling mechanism in the electrode array is discussed. We propose a silicone rubber–hydrogel actuator where the hydrogel forms a percolating network in the dorsal silicone rubber compartment of the electrode array to exert bending forces at low volume swelling ratios. A material study of suitable polymers (medical-grade PDMS and hydrogels), including parametrized bending curvature measurements, is presented. The curvature radii measured meet the anatomical needs for positioning electrodes very closely to the modiolus. Besides stage-one biocompatibility according to ISO 10993-5, we also developed and validated a simplified mathematical model for designing hydrogel-actuated CI with modiolar hugging functionality.

## 1. Introduction

Cochlear Implants (CIs) are currently the best solution in compensating for sensorineural hearing loss by direct electrical stimulation of the auditory nerve cells in the inner ear [1]. CIs consist of electrode shafts made from silicone rubber with platinum electrode contacts that are connected via platinum wires with the receiver–stimulator of the implant [2]. Even though this implant is the gold standard for sensorineural hearing loss, a series of limitations, e.g., listening to music or communicating in noisy environments, are yet to be overcome [3,4]. One reason for these limitations is the low effective number of stimulating channels [5], which is due to the wide distance between electrodes and the nerve ganglion cells to be stimulated [6]. Since these nerve cells are situated in the modiolus, a reduction in the distance between implant and cells can be achieved using a CI electrode bent towards the modiolus. Available products are pre-bent shafts that are released from straight stylets during insertion, known as the Contour Advance^®^ (CI612) electrode shaft from the Cochlear Nucleus Profile™ (Cochlea Ltd., Sydney Australia), the Hi-Focus electrode (Advanced Bionics, Valencia USA), and the peri-modiolar push-wire-based electrode Combi 40PM (MED-El, Innsbruck Austria) [5,7]. Another research approach involves using smart alloys which, in contact with tissue at body temperature, change from a stretched shape into a previously impressed curved shape [8]. However, implanting these smart alloys as straight electrodes was obstructed by the reaction of the immediate materials to, e.g., body heat (surgeon) or other heat sources in the operating room, such as lamps.

To overcome the distance between the electrodes and the modiolus, the integration of an actuator into CI arrays using the swelling properties of hydrogels might present a promising alternative.

### 1.1. Fundamentals of Material and Components

Polydimethylsiloxanes (PDMS) are silicone elastomers used in medical applications since their material properties, e.g., flexibility, biocompatibility, stability, hemocompatibility, and sterilization resistance, are advantageous for longtime implantation. For neuronal implants, liquid silicone rubbers (LSR) or room-temperature polymerized silicone rubbers (RTV) are typically used [9,10] due to favorable characteristics for implants, such as a high degree of physical or chemical purity and thus a very low negative tissue response [11]. PDMS has proven to be a suitable material for integrations in manufacturing CI-electrode arrays.

Hydrogels are three-dimensional hydrophilic crosslinked polymer networks able to swell and shrink with specific characteristics of deformation. They are increasingly used in biomedical applications [12] as mechano-active implants, for example, for tissue repair and regeneration, such as arthrosis, by using mechanical stimuli in order to control cell or drug delivery, accelerating tissue remodeling or healing processes [13]. Examples of synthetic hydrogels are crosslinked poly(methacrylic acid), poly(vinyl alcohol), and polyacrylamide (PAM) [14]. The first silicone rubber–hydrogel composite in the context of CIs under investigation was polyacrylic acid (PAA), used as filler material in a silicone rubber called Silastic. In vivo and in vitro biocompatibility tests showed positive cellular and tissue responses, and an intracochlear curled array could be achieved [15]. Abbasi et al. continued developing an intra-cochlear electrode by using PDMS-PAA as interpenetrating polymer networks proven to have hydrophilic characteristics. Positive cytotoxicity tests were shown, but significantly different cell-adhesion behavior was observed. The best adhesion was achieved for composite samples consisting of 20% (*w/v*) PAA content, which was suggested to result from an increased wettability of the PDMS-PAA network [16]. To increase the possibility of usage in vivo, our investigation into this field was completed using medical-grade silicone rubber.

In this study, we compare the swelling behavior of medical-grade silicone rubbers Nusil Med 4850 (Avantor Sciences, Radnor, PA, USA) [17] and Silpuran 2430 (Wacker Chemie AG, München, Germany) [18] as well as the hydrogel polyacrylamide/acrylate (PAMAA) as a swelling filler as an approach towards a curved CI electrode array. PAMAA is a hydrophilic copolymer of acrylamide and acrylic acid but crosslinked enough to be insoluble in water. It can absorb up to 400% of its own weight in water and swells proportionately [19]. Based on our previous work presented by Stieghorst et al., the general feasibility of the actuating effect with hydrogel–silicone rubber composites was shown by using the PDMS Sylgard 184 and the hydrogel PAM [20,21,22]. Due to further experimental results with PAM that negatively influenced the stability of manufactured electrode arrays, we extended the concept by testing different material combinations with the hydrogel PAMAA in different sample designs. In this context, the usability of two medical-grade PDMS (restricted: Silpuran 2430; unrestricted: Nusil Med 4850) in combination with PAMAA is shown in the present study. The general principle of the curling and hugging actuation results from the use of a bimorph actuator consisting of a pure silicone rubber shaft containing electrodes and wires and a silicone rubber–hydrogel composite attached on top of the electrode shaft. The dehydrated hydrogel particles that are compounded into the silicone rubber may swell under water uptake, which leads to the desired curling, as reported by Walling et al. [23]. In our approach, the Ringer solution was used as a substitute for human perilymph [24,25]. The experiments were designed to evaluate the biocompatibility and swelling behavior as well as the functionality of the actuator in CI-like samples.

### 1.2. Fundamental Physical Processes

This section derives a general description of bending actuation based on swelling tests and visual inspection of the bent test specimen. For the free swelling tests, hydrogel–PDMS composite samples were used, as described in Section 2. For this bending study, bimorph actuators, as given in design B of the same figure, were considered. From literature and data sheets, the following parameters were available: the Young’s modulus of the pure silicone rubber (E_PDMS_) and its density (ρ_PDMS_), the density of the dry hydrogel (ρ^H, dry^), and the weight fraction of the dry hydrogel contained in the silicone rubber matrix (f_H_). As the considerations presented in the following sections only address the final steady states, we do not take the particle size into account, which has an influence on the dynamic behavior.

The measurable quantities obtained by visual inspection are the thickness of the PDMS layer in design B (h_PDMS_) and the corresponding thickness of the composite layer in its dry, initial state (h_comp,init_). After swelling, the quantity h_comp,init_ reaches the h_comp,fin_ value according to the newly swollen state. The bending radius R of such a swollen test specimen may also be assessed by visual inspection and fitting. The swelling process can be described by analogy with osmosis, where the hydrogel acts as both a semi-permeable membrane and receptacle. The osmotic pressure in the hydrogel is limited by the mechanical stress corresponding to the maximum elastic deformation of the PDMS network during hydrogel swelling, which must be balanced with the external osmotic pressure of the perilymph/Ringer solution [26]. The amount of swelling can be calculated from the measurements of the Ringer solution uptake of the free swelling composite samples (design A), with m_i_ indicating the initial weight of the sample in a dry state and m_f_ indicating the final weight of the same sample in a swollen state.

The physical bending model is based on the following considerations. It is assumed that in the free-swollen composite material in specimens of design A, the effective net force is zero. Actually, the internal osmotic pressure of the hydrogel phase in a swollen composite sample is balanced by the opposite stress generated by the elongation of the interweaving PDMS matrix. In contrast, the PDMS–hydrogel composite material in design B will have regions of mechanical limitation of swelling. In particular, in the regions near the interface of the PDMS/composite layer, an effective pressure will predominate, which arises from the internal osmotic pressure exceeding the reduced elongation of the PDMS but is balanced by the remaining elongation induced into the pure, adjacent PDMS layer. The distribution of this internal pressure from the PDMS/composite interface towards the freestanding end of the composite is initially unknown and must be clarified by additional investigations.

In order to make stress and pressure accessible, the Ringer solution uptake of the composite is transformed into the corresponding volumes. Starting from the known quantities *m_i_, m_f_,* and *f_H_*, the volumes *V_i_* and *V_f_* can be calculated via the densities of the PDMS (*ρ_PDMS_*) and the hydrogel phase in its dry (*ρ_H,dry_*) and swollen state (*ρ_H,sw_*)

(1) $$V_{i}=m_{i}\left(\frac{1-f_{H}}{\rho_{PDMS}}+\frac{f_{H}}{\rho_{H,dry}}\right)$$

(2) $$V_{f}=\frac{m_{i}\left(1-f_{H}\right)}{\rho_{PDMS}}+\frac{m_{f}-m_{i}\left(1-f_{H}\right)}{\rho_{H,sw}}$$

which yields the relative volume expansion *ν* (expressed as (*V_f_-V_i_)/V_i_*):

(3) v=1−fHρPDMS+mfmi−1+fHρH,sw1−fHρPDMS+fHρH,dry

Due to the bending of the test specimen, we assume a radially increasing value of the relative volume expansion *ν(r)*.

In a free swelling PDMS–hydrogel composite, the swelling is always isotropic, which means that the linear expansion factor $\Delta l/l_{0}=\sqrt[{3}]{\nu }$ is the same irrespective of the spatial direction; we have to check this for the complex stress and pressure balance in design B. However, it might be reasonable to expect that in the vicinity of the interface from the composite to the surrounding fluid, almost free swelling is reached.

**Figure 1:**
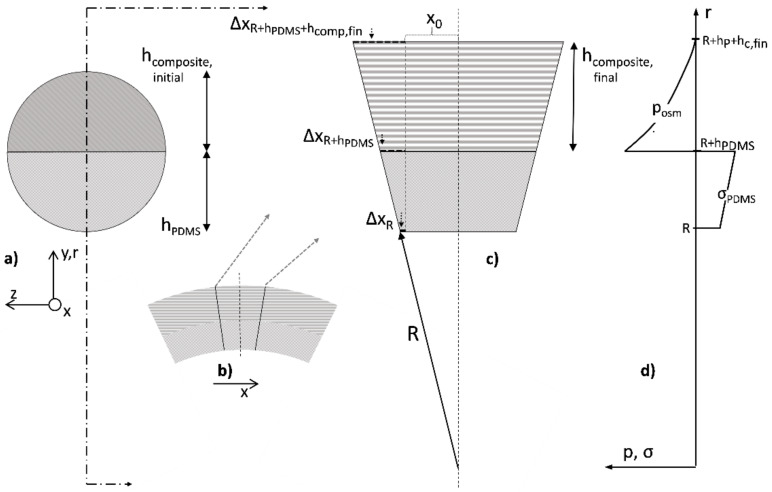
Swelling of cylindrical specimens “Design B”: (**a**) cross-section with pure silicone rubber (gray) and dry, initial silicone–hydrogel composite (hatched) compartments, (**b**) cross-section showing bending in (x-) direction due to swelling of the silicone–hydrogel composite, (**c**) calculation of elongations in x-direction for given bending radius R and final height of the composite layer and (**d**) schematic distribution of stress and pressure in PDMS and composite compartments.

An approach with finite differences is sketched in Figure 1. On the upper left side, a cross-section of design B is given. The bending here takes place out of the drawing plane along the *x*-axis. The small inset shows that bending along the section plane and a small segment of this circular bending was magnified and related to the bending radius *R*. This illustrates that the bending of the PDMS composite is different from a classic bimorph, as both layers undergo elongations *Δx_i_,* as can be seen in Figure 1. As indicated in the graph to the right, the stress σ in pure PDMS must increase from the fluid–PDMS interface (position *R*) towards the internal PDMS composite interface (position *R* + *h_PDMS_*). In the swollen composite material, on the other hand, it seems to obvious that there is an osmotic pressure p that decreases towards the composite–fluid interface (position *R* + *h_PDMS_* + hcomp,fin). Thus, the elongations along the X-axis of the composite in its swollen state are *Δx_R_, Δx_R+hPDMS_,* and *Δx_R+hPDMS+hcomp,fin_*, as graphically illustrated in Figure 1c. From the similar triangles characterized by the hypotenuses denoted by *R, R + h_PDMS_*, and *R + h_PDMS_* + *h_comp,fin_*, the following equalities result:

(4) $$\frac{x_{0}+\Delta x_{R}}{R}=\frac{x_{0}+\Delta x_{R+hPDMS}}{R+h_{PDMS}}=\frac{x_{0}+\Delta x_{R+hPDMS+hcomp,fin}}{R+h_{PDMS}+h_{comp,fin}}$$

Equation (4) can be rewritten as:

(5) $$R=\left(1+\varepsilon \left(R\right)\right)\frac{R+h_{PDMS}}{1+\varepsilon \left(R+h_{PDMS}\right)}=\left(1+\varepsilon \left(R\right)\right)\frac{R+h_{PDMS}+h_{comp,fin}}{1+\varepsilon \left(R+h_{PDMS}+h_{comp,fin}\right)}$$

where *ε* signifies the extensional strain or elongation at different interfaces of the composite expressed as follows: *ε(R)* = Δ*x_R_/*Δ*x_0_, ε(R + h_PDMS_)* = Δ*x_R_ + h_PDMS_/*Δ*x_0_ and ε(R + h_PDMS_ + h_comp,fin_)* = Δ*x_R_ + h_PDMS_ + h_comp,fin_/*Δ*x_0_*. At the same time, it is assumed that an isotropic swelling at the composite–fluid edge can provide the necessary boundary condition for the determination of all further parameters. This holds if shear moduli and transverse contractions can be neglected, which leaves the sectional planes in the radial direction free of forces. Furthermore, if the modulus of elasticity of the hydrogel in its swollen state makes a negligible contribution to the Young’s modulus of the composite body compared to the PDMS, one obtains *E_comp,fin_* *≅* *(1-f_H_)(ρ_PDMS_/ρ_H,dry)_* *E_PDMS_* for the freely swollen composite layer. Under the variable volume expansion ν(r), this leads to:

(6) $$E_{comp,sw}\left(r\right)=E_{PDMS}\frac{1-f_{H}}{\nu \left(r\right)}\times \frac{\rho_{PDMS}}{\rho_{H,dry}}$$

Basically, both the stress in the PDMS and the pressure in the composite act like torques on a one-armed lever with a pivot point at the center of the bending circle. These both sum to zero, as otherwise, the pivoting point would become displaced. While the torques for the PDMS layer can be easily specified, the pressure curve in the composite actually requires the functional for equilibrium pressure via the volume-limited swelling of the hydrogel. In this generalized form, one obtains the final relationship:

(7) $$0=\left(R+\frac{h_{PDMS}}{2}\right)\frac{\varepsilon \left(R\right)+\varepsilon \left(R+h_{PDMS}\right)}{2}E_{PDMS}-\int_{h_{PDMS}}^{h_{comp,fin}}p\left(r,\varepsilon \left(r\right)\right)dr$$

## 2. Materials and Methods

### 2.1. Sample Preparation

Two silicone rubbers were used to evaluate their suitability for the envisioned application. Silpuran 2430 (Wacker Chemie AG, Burghausen, Germany) is a two-component (RTV2) silicone rubber approved for human use in a restricted interval not exceeding 28 days. The two-component medical LSR NuSil^®^ MED-4850 (Avantor Sciences, Radnor, PA, United States) is approved for use in human implantation for a period of greater than 28 days. The components of both PDMS are mixed in a ratio of 1:1. Relevant PDMS properties are shown in Table 1. Nusil Med 4850 has a tensile strength of 10.17 MPa and a maximum elongation of 675%, while Silpuran 2430 has a tensile strength of 6 MPa and a 540% elongation. Therefore, mechanical stability after the expansion is expected for both PDMSs [17,18].

**Figure 2:**
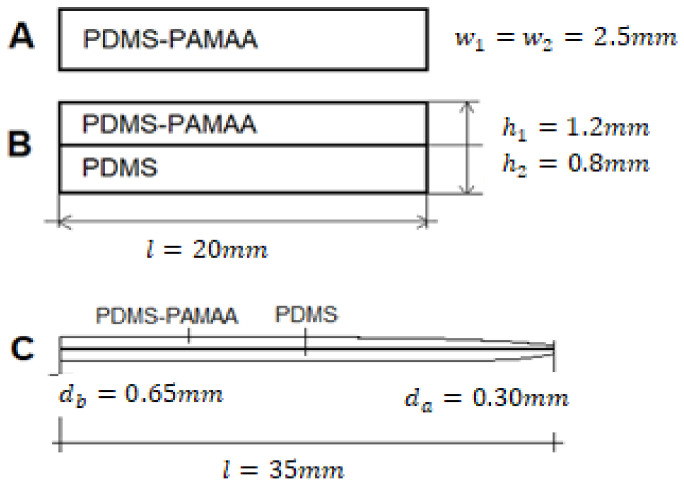
Schematic of the three sample designs, where w, h, and l are the width, height, and length of the outlined samples, respectively. (**A**): Rectangular design consisting of one-layer composite compound material; (**B**): rectangular piggy-back design: the bottom layer is the silicone rubber, and the upper layer is the compound material; (**C**): CI-shape design with an additional layer of composite material.

Samples consist of either Silpuran 2430 or Nusil Med 4850 mixed with the hydrogel powder (polyacrylamide/-acrylate (PAMAA) (AC33-GL-000110, Goodfellow, Hamburg, Germany)). Three sample designs were used for the investigation (see Figure 2). Sample design A was used to investigate basic material properties. Sample designs B and C were exclusively produced with the unrestricted medical LSR Nusil Med 4850 and were used for proof-of-principle observations. For samples B and C, the compound was added onto a silicone rubber layer to investigate the interactive material behavior in the course of the swelling process and to evaluate the compounds’ readiness to use as an actuator on CIs. The sample design A and B size (see Figure 2) results in a surface-to-volume ratio of 1.7 mm, which is close to that of CI electrode array, as can be seen in Figure 2. Sample design C had measures in accordance with cochlea measurements. The molds for sample designs A and B were 3D-printed (Formlabs, Berlin, Germany) with artificial resin (Clear resin, Formlabs, Berlin, Germany). The mold for sample type C was produced manually with acrylic glass.

Since the grain diameter is 2.5 mm, PAMAA granules were ground and sieved into different grain-size fractions to evaluate the influence of grain size on the actuator’s performance (see Appendix A.1). Three grain-size fractions were produced using a ball mill (Pulverisette 23, Fritsch, Idar-Oberstein, Germany). The first fraction contained all grains with a diameter (∅)≤ 20 µm, the second fraction consisted of 20 µm<∅≤50 µm (diameter) grains, and the third fraction contained 50 µm<∅≤100 µm (diameter) grains. The particle size distribution of these fractions was determined statistically using a helium-neon laser for optical spectrometry (HELOS) sensor (Sympatec GmbH, Clausthal-Zellerfeld, Germany).

In preliminary tests, several PAMAA contents from 10 wt% up to 40 wt% were investigated. Samples with initial hydrogel percentage of >30 wt% exhibited very high volume expansions, unfit for the dimension in the inner ear. Therefore, this study focused on PAMAA hydrogel fractions between 20 and 30 wt%, as given in Table 2. Silicone rubber and hydrogel particles were mixed together by means of a speed mixer (Hauschild Engineering, Hamm, Germany) at 3500 rpm. As displayed in Table 2, sample designs B and C were not prepared with the smallest grain-size fractions. This was the result of the low amount of the respective powder after long grinding and sieving processes. It was decided to first evaluate the other fractions. Sample design C was then only prepared with the medium grain-size fraction due to the results from sample design A.

### 2.2. Swelling Tests

To investigate the hydrogel swelling, samples were stored in Ringer solution for up to 28 days in a dry cabinet at 37 °C. Test containers (microboxes made of PP) were filled with 10 mL Ringer solution (Berlin-Chemie AG, Berlin, Germany), then dry samples were added after measuring the initial weight. The samples’ weight change was measured eight times in one-hour steps after initial weighing. Then, measurements were completed in 24 h steps for seven days, followed by one measurement per week. Before weighing, samples were dabbed lightly on a tissue wipe to remove any adhering liquid drops. The swelling ratio was plotted vs. the square root of time. For sample designs B and C, the values of radii of each sample and their corresponding median were determined using GraphPad Prism.

### 2.3. Biocompatibility Tests

For first biocompatibility information on the silicone rubber–hydrogel, the water-soluble tetrazolium dye assay (WST-1 assay, Cell proliferation Reagent WST-1, Roche GmbH, Basel, Switzerland) was used in accordance with EN ISO 10993-1-19. Further information can be found in Appendix A.2. If the cell viability was confirmed, consecutive tests need to be performed in later steps of the conformity-evaluation procedure.

## 3. Results and Discussion

### 3.1. Hydrogel Processing and Particle Size Distribution

Hydrogel with an initial particle size of 2.5 mm was ground and sieved to generate three particle-size fractions. Several iterations of the grinding process were necessary to obtain sufficient material for *n* = 6 samples for each of the three weight percentages under evaluation (20 wt%, 25 wt%, 30 wt%). Only small amounts of particle size fraction <20 µm were produced due to rapid reagglomeration of the hydrogel particles.

Optical spectroscopy (laser scattering/diffraction) was performed to determine the particle size distribution in the investigated grain-size fractions. Both cumulative and density distributions were calculated. For each sample, three measurements were taken that showed varying median values of particle sizes depending on the sieve mesh size.

**Figure 3:**
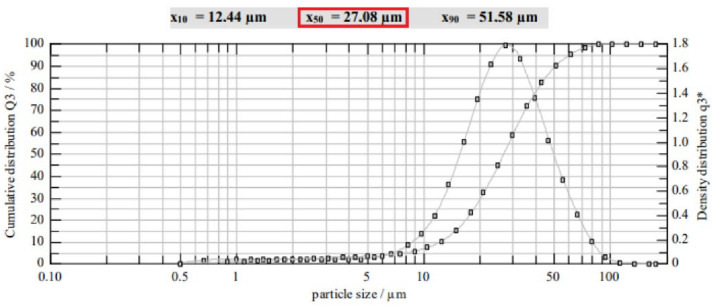
Exemplary distribution diagram of mean particle size distribution measured via spectroscopy (laser scattering) showing cumulative and density distributions for one sample of the grain-size fraction <20 µm. Measurement was repeated three times (see Table 3).

Figure 3 shows a distribution diagram with both cumulative and density distributions obtained through laser diffraction for hydrogel powder of the grain-size fraction <20 µm.

After sieving with a mesh size of 20 µm, the particle fraction was expected to consist of particles <20 µm, while spectroscopy results showed a median for one sample of 27.08 µm (marked red). As can be seen in Figure 3, a large number of particles with higher grain size was detected, while a lower quantity of particles with grain size smaller than <20 µm was recorded. Particle sizes as median and standard deviation values averaged over three measurements are listed in Table 3. With the exception of the smallest fraction, the PAMAA has median values that correspond to the grain-size mass fractions expected after sieving. In the following, the ranges of the grain size distribution are replaced by the measured grain-size fraction median values of 77 µm (replacing 50–100 µm), 48 µm (replacing 20–50 µm), and 28 µm (replacing 0–20 µm).

It is known that the process of mixing silicone rubber with crosslinked fillers in elastomers requires the break-down of the pellets for efficient dispersion of pellet fragments as small micro-scale particles [27]. The smallest possible grain-size fraction proposed in our approach was 20 µm, with a measured median value of 28 µm. Assuming the 20 µm sieve is flawless, this result confirms a particle aggregation after the sieving procedure. During hydrogel manufacturing, reagglomeration is a known problem caused by, e.g., residual water [28]. Reagglomeration is likely to occur due to water uptake of the hydrogel particles in our processing and storage environment. Yet, the agglomeration mainly seems to occur for particle sizes <20 µm. Yanagioka et al. reported that the agglomeration of inorganic particles in polymeric composite materials is fundamentally important but not yet completely understood [29]. Their study also reported a relationship between particle distribution within a polymer matrix and some composite mechanical properties, such as agglomeration and swelling [29].

### 3.2. Swelling Tests

#### 3.2.1. Design A—Hydrogel–Silicone Rubber-Compound Properties

**Figure 4:**
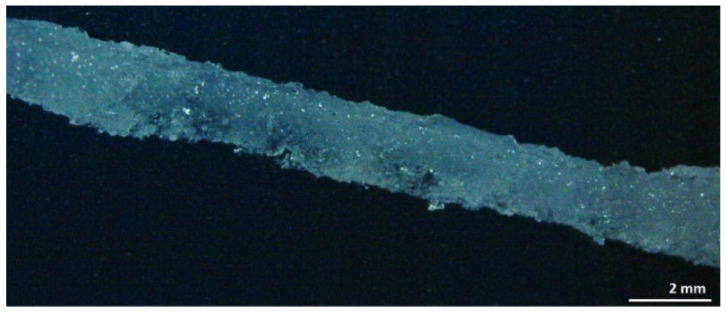
Optical microscopy image of sample design A representing Nusil Med 4850-PAMAA in swollen state (672 h). Hydrogel particle size fraction was 77 µm, and hydrogel content was 30 wt% prior to swelling.

**Figure 5:**
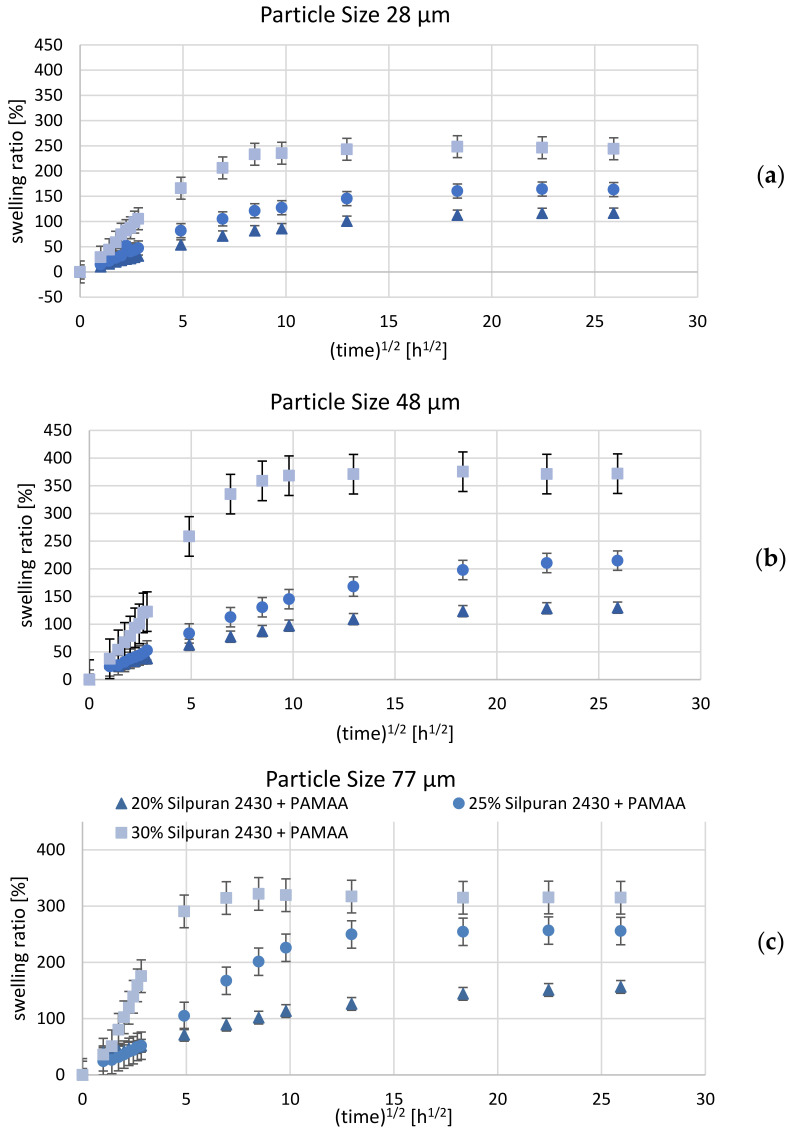
Mean swelling ratio over square root of time (total swelling time: 672 h) for Silpuran 2430-PAMAA composite samples with initial hydrogel content of 20 wt%, 25 wt%, and 30 wt%. Error bars indicate standard deviation for test samples with hydrogel particle size (median) of (**a**) 28 µm, (**b**) 48 µm, and (**c**) 77 µm. For each material combination, *n* = 6 samples were investigated.

The sample weight measurements indicate a rapid mass increase during the first 24 h after immersing the sample in Ringer solution. An increase in hydrogel concentration induces a significant increase in the degree of swelling, which is, however, influenced by the PDMS matrix. Figure 4 shows a sample of design A, which exhibits a typically uneven surface structure after the swelling process. This structure is due to inhomogeneous particle distribution in the initial compound. Based on the fact that swelling behavior is dependent on the hydrogel fraction, the crosslinking density, and the particle grain size [30], three parameters were evaluated in our study, including the influence of the silicone rubber matrix, the initial hydrogel content, as well as the hydrogel particle size, and will be discussed in the following. In general, the samples showed a rapid weight increase during the first 24 h after being placed in the Ringer solution. PAMAA-Silpuran 2430 samples all show a rapid water uptake in the first 24 h. Regarding the swelling characteristics, samples with the highest particle content (30 wt%) dispersed into Silpuran 2430 show the highest volume swelling ratio (max. 370%), while the compound with the lowest amount of dry hydrogel (20 wt%) led to the lowest volume swelling ratio (max. 156%) (for comparison, see Figure 5). For the initial hydrogel percentage of 20 wt% and 25 wt%, the maximum volume swelling increases with an increase in grain size. Thus, for an initial hydrogel amount of 25 wt%, the highest results are 164% (28 µm mean grain size), 214% (48 µm mean grain size), and 256% (77 µm mean grain size). However, this fact could not be observed for the 30 wt% hydrogel. For this initial hydrogel amount, the highest volume swelling ratio was obtained for a grain-size fraction of 48 µm. The grain-size fraction influences the duration of water uptake until the saturation point is reached.

Nusil Med 4850-PAMAA samples showed a steep increase in swelling ratio over the first hours of tests until they reached a saturation point. After this point, the swelling evolved much slower and mainly depended on the hydrogel particle size. Overall, for the smallest and the largest grain-size fractions, the swelling ratio was leveled down around the saturation point. In contrast to the compounds made of Silpuran 2430, the smallest grain-size fraction contained in the Nusil Med 4850-PAMAA composite led to the highest swelling ratios (187%/30 wt% PAMAA), and the highest grain-size fractions (77 µm) led to the lowest maximum swelling ratios (113%/25 wt%). In the case of hydrogel particles with a mean grain size of 48 µm, the swelling ratio followed a descending tendency to an equilibrium value after reaching the saturation point. For the smallest and medium grain-size fractions of hydrogel, an increased amount of PAMAA led to an increased swelling ratio. This was not true for the samples containing the highest grain-size fractions, where the highest initial weight percentage of PAMAA exhibited the lowest swelling ratio.

**Figure 6:**
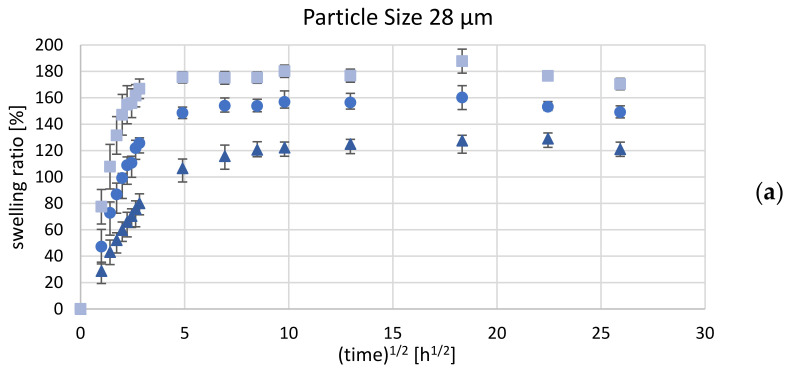

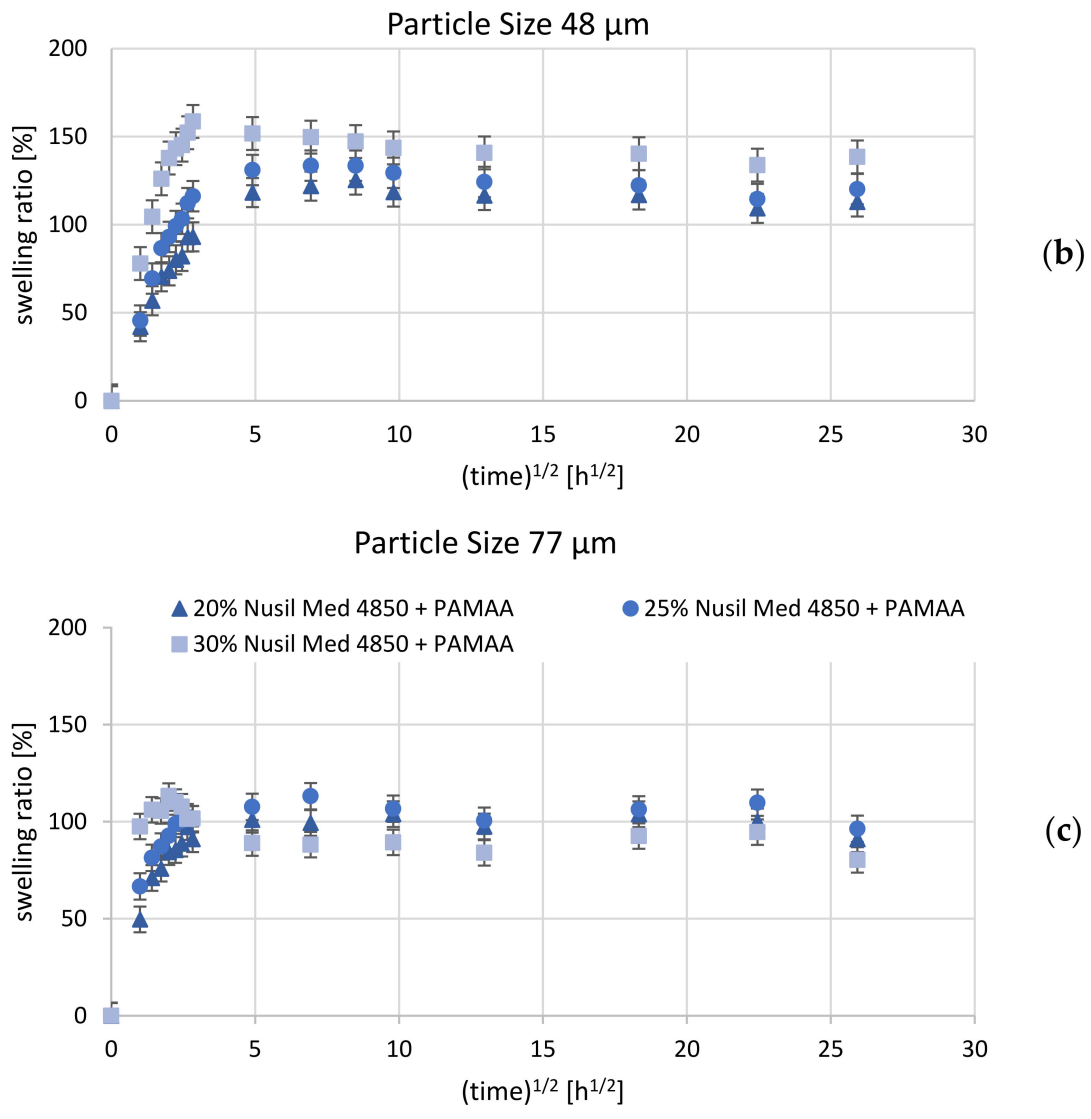
Mean swelling ratio over square root of time (total swelling hours: 672) for Nusil Med 4850-PAMAA composite samples with initial hydrogel content of 20 wt%, 25 wt%, and 30 wt%. Error bars indicate standard deviation for test samples with hydrogel particle size (median) of (**a**) 28 µm, (**b**) 48 µm, and (**c**) 77 µm. For each material combination, *n* = 6 samples were investigated.

For test results with Silpuran 2430-PAMAA, the lowest grain-size fraction of 28 µm and the highest fraction of 77 µm displayed lower swelling ratios compared to the middle grain-size fractions with 48 µm particles. However, this trend cannot be observed for tests with Nusil Med 4850-PAMAA. Here, the lowest grain-size fraction indicated the highest swelling ratios, while the middle and greatest grain-size fractions had similarly low swelling ratios. As can be observed for several material combinations (see Figure 5 and Figure 6), the swelling ratio decreased after reaching equilibrium. This is assumed to be due to particle loss. In general, the Nusil Med 4850-PAMAA composite samples achieved a lower equilibrium swelling compared to samples composed of Silpuran 2430 (see Figure 5 and Figure 6) for each grain-size fraction. The time until a saturation point was reached differed significantly for both silicone rubbers, irrespective of the amount of hydrogel or grain-size fraction. A possible explanation goes in parallel with the leaching as seen above: Nusil has a weaker interaction with the hydrogel, which speeds up the water uptake. Silpuran holds the particles tighter, which slows down the swelling. This indicates that the experiments can be modeled with the described equations in Section 1.2. Another difference in the swelling behavior between the silicone rubbers is that PAMAA with Silpuran 2430 exhibits higher swelling ratios than PAMAA with Nusil Med 4850. In the context of sodium polyacrylate, the dependence of the shear modulus on swelling behavior results in an ascending tendency. Additionally, the osmotic swelling pressure and the elastic modulus strongly depend on the ionic composition of the surrounding fluid [26]. Among many things, the Young’s modulus and Shore hardness seem to influence the swelling ratio. Our performed swelling measurements show the following: the Nusil Med 4850 (hardness Shore 50)-based composites displayed lower swelling ratios compared to those obtained for Silpuran 2430-based hydrogels, for which the rubber component was characterized by a lower Shore hardness (20) and, consequently, by an augmented elasticity. This leads to the conclusion that increased Shore hardness leads to decreased elasticity and, therefore, to reduced hydrogel swelling in the network.

#### 3.2.2. Design B—Bimorph Hydrogel–Silicone Rubber-Composite on Silicone Rubber Base

**Figure 7:**
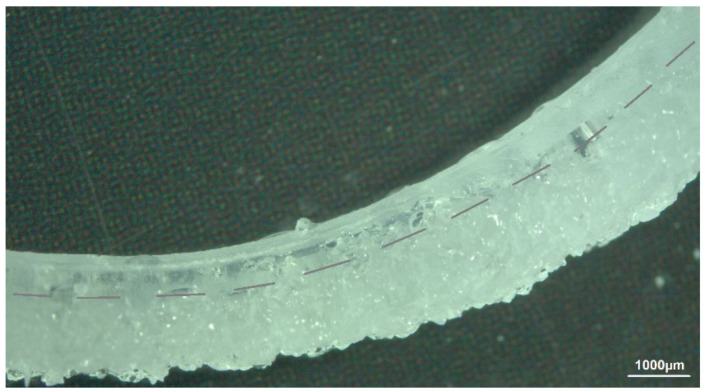
Optical microscopy image of swollen sample design B using Nusil Med 4850-PAMAA: swelling medium—Ringer solution; swelling time—168 h; hydrogel particle size fraction—77 µm; initial hydrogel content—20 wt%.

**Figure 8:**
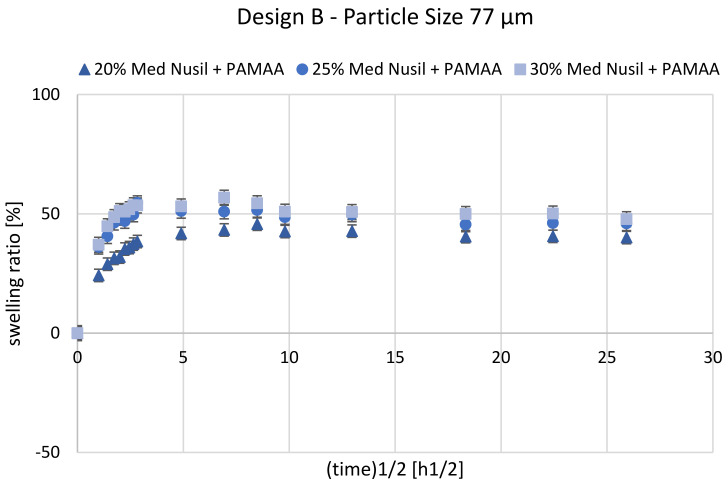
Mean swelling ratio over square root of time (total swelling time: 672 h) for Nusil Med 4850-PAMAA composite design B samples with initial hydrogel concentration of 20 wt%, 25 wt%, and 30 wt%. Error bars indicate standard deviation for test samples with hydrogel particle size (median) of 77 µm. For each material combination, *n* = 6 samples were investigated.

The casting mold for manufacturing samples with design B proved to be difficult since the composite layer had to be applied onto a layer of pre-vulcanized silicone rubber. Nevertheless, the resulting interface proved to be mechanically stable enough to withstand delamination after water uptake of the hydrogel phase (see Figure 7). Figure 8 shows the mean swelling ratio as a function of the square root of time for Nusil Med 4850-PAMAA samples with a grain-size fraction of 77 µm.

In comparison to samples of design A, the samples of design B displayed a distinctly lower swelling ratio with a Ringer solution absorption, attaining a maximum at a relatively early stage of swelling and then slightly deswelling until equilibrium was reached. The swelling ratio of sample design B with an initial hydrogel content of 30 wt% did not reach 60% during the first 24 h. In the course of the next measurements until 336 h, the maximum value of the swelling ratio declined by up to 45%. Samples with 25 wt% PAMAA reached a maximum swelling ratio of 55% after 8 h, and Ringer solution saturation was detected after 336 h at 45%. Samples with 20 wt% hydrogels have a maximum swelling ratio of 45% after 72 h. After 336 h, saturation was measured at 41%. Compared to the results of design A with the same material composition and grain size, a reduction in half of the swelling ratio from 100% to 56% was found. In general, the lower swelling ratios were caused by the silicone rubber’s retarding force given by an additional PDMS layer, which is the solid part within the process for inducing the matrix bending apart from swelling. This is also confirmed and was expected according to the theoretical description of the swelling process in Section 1.2.

**Figure 9:**
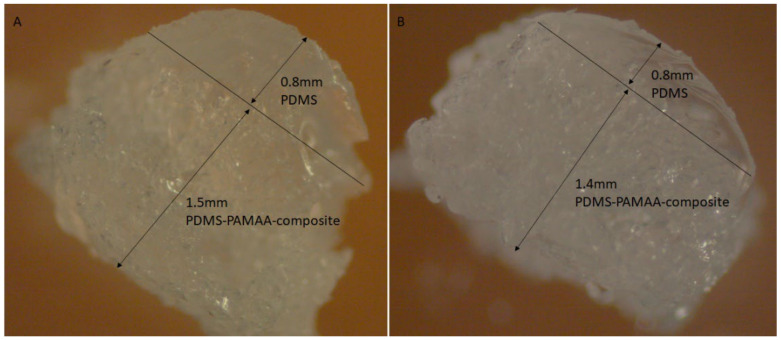
Cross-sections of design B samples with (**A**) initial hydrogel content of 20 wt% and a hydrogel particle size of 48 µm, and (**B**) initial hydrogel content of 30 wt% and a hydrogel particle size of 77 µm.

To evaluate the swelling calculations mentioned in Section 1.2, cross-sections of samples of design B were evaluated and measured, as seen in Figure 9.

Based on the considerations in Section 1.2, a computational model was set up in which all known parameters were first included. These were the density of the silicone elastomers *ρ_PDMS_* = 0.965 g/mL and their Young’s modulus of *E_PDMS_* = 6 MPa, as well as the density of PAMAA in the dry state *ρ_H,dry_* = 0.75 g/mL. The density of PAMAA in the swollen state reaches that of water *ρ_H,sw_* = 1.0 g/mL. For further comparison, data on free-swelling Nusil specimen A were evaluated and converted to volume increases. The mass increases *m_f_/m_i_*, which is in the range of 2.0–2.8, were thus transferred to enlarged volumes towards 149%–198%. A rough comparison of the linearly achieved elongations in design B (see Figure 9) shows rather larger values compared to those resulting from the measurements with specimen A. From this, it was concluded that free swelling of specimen B at the free end of the composite layer (*h_comp,fin_*) was achieved with high probability, and therefore the main working hypothesis of the approach in Section 1.2 holds. Thus, for the specimen shown in Figure 9a (*f_H_* = 20%, 48 µm size fraction) with *h_PDMS_* = 0.8 mm and *h_comp,i_* = 1.2 mm or *h_comp,fin_* = 1.5 mm, a radius of curvature of 7.6 mm was achieved. The elongation at the free end of the composite *ε (R + h_PDMS_ + h_comp,fin_)* was subsequently found to be 0.20 and was very close to the value of 0.186 obtained from the associated data of the A designs. The corresponding Young’s modulus of the swollen silicone–hydrogel composite averaged *E_comp,sw_* = 3.7 MPa. The mean pressure in the swollen hydrogel composite was thus 0.68 MPa. The mean stress in a layer of pure PDMS was 0.97 MPa. The stress in the PDMS composite interface reached a value of 1.67 MPa, assuming a linear pressure profile in the composite.

This very consistent picture achieved with the 48 µm particles in design B is no longer reached for the 77 µm fraction. For *h_comp,fin_*, only 1.4 mm is measured, which means that the elongation of 0.14 was below the maximum value of 0.15 from the measurements with design A. Additionally, the bending radii reaching 14 mm indicated the partial detachment of the hydrogel. Nevertheless, the calculated stress along the inner interface still reached values of 1.2 MPa.

#### 3.2.3. Design C—CI Shape with Silicone Rubber–Hydrogel Top Layer

**Figure 10:**
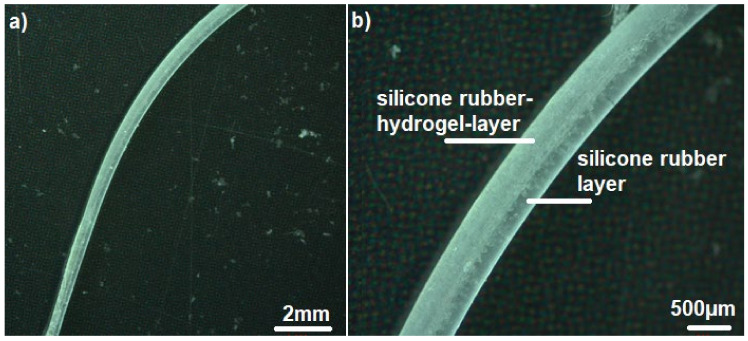
(**a**) Optical microscopy images of sample design C of Nusil Med 4850-PAMAA (25 wt% hydrogel particle size fraction of 48 µm) in dry state deposited on Nusil Med 4850 imitating a CI. (**b**) is an enlargement of image (**a**).

**Figure 11:**
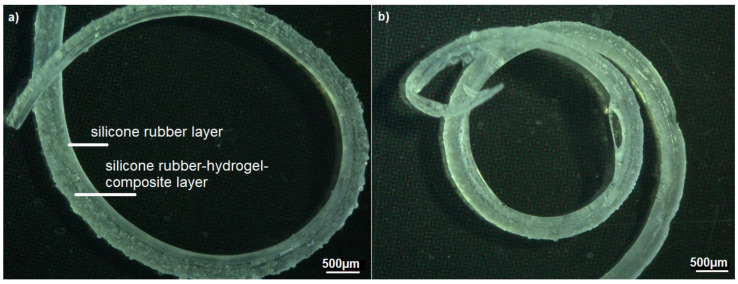
Optical microscopy images of two samples of design C manufactured with Nusil Med 4850-PAMAA (20 wt% hydrogel particles; grain-size fraction 48 µm) deposited on Nusil Med 4850 as a substrate. Samples are shown after (**a**) 168 h and (**b**) 336 h in Ringer solution.

Figure 10 shows a sample consisting of Nusil Med 4850-PAMAA in a dry state, while Figure 11 displays two examples of Nusil Med 4850-PAMAA after 168 h and 336 h of swelling in Ringer solution, proving excellent interlayer adherence. The samples proved to be mechanically stable even after a total swelling time of 672 h. The expected curving was achieved. The achieved curvature radius for one sample of design C (Figure 11b) was 3.9 mm for the basal and middle section and 3.1 mm for the apical section. The determined radii for all samples are listed in Table 4.

The volume swelling ratios of design C being lower than those of sample design A was likely due to the same effect as for sample design B. However, the swelling ratios of the CI shapes (about 40%) and the samples of design B (between 40 and 55%) achieved quite similar swelling values. As listed in Table 4, the determined radii of samples of design B (10.9–16.6 mm) are considerably higher than those of the samples of design C (between ~2.5 and 3.9 mm). This is assumed to be due to the different sample geometries. Samples of design B consist of a PDMS layer with a significantly higher thickness and therefore stiffness, which inhibits the curvature. In contrast, the CI-shape design facilitates an increased curvature due to its thinner PDMS layer and thin diameter towards the apex of the array.

In design C, only two test samples were fabricated with 20% hydrogel content in the 48 µm particle size fraction. In the Ringer solution, both samples showed 360° full-circle bending after 168 h and even achieved two complete turns after 336 h. One of the samples reached a mean bending radius of 3.6 mm and showed a loss of swollen layer thickness of about 100 µm in the visual inspection. The second sample, on the other hand, achieved a mean bend radius of 1.9 mm and the best value of 1.25 mm in the middle range. At 300 µm PDMS thickness and initial composite thickness of 350 µm in the basal region, the linear swell increase was 17%, and the corresponding isotropic volume increase was 60%, respectively. The elongation at the free-swelling end was 14.6%, well below the theoretically expected value of 18.6%. The stress values were 0.63 MPa on average, 1.1 MPa at the inner interface, and the mean pressure prevailing in the composite was 0.49 MPa. Overall, these values were very similar to the test specimen described above according to Design B with a 77 µm particle size fraction. That is, a loss of hydrogel particles may still have occurred here as well. Overall, the achievement of two full circles exceeds the minimum requirements of 1.5 revolutions or 540° as established for CI. The local achievement of a 1.25 mm bending radius would fit even the narrowest apical turn in the human cochlea, whose radius is about 1.3 mm. Even though the number of specimens is statistically small and no further parameter variations are available, it is clear that as the specimen dimensions decrease, the bending radii also decrease while the internal stress and pressure ratios remain the same. The possibility that leaching also occurs in the 48 µm particle size fraction with thinner coatings requires further investigation.

In addition, the observed volume increase in such actuating implant shafts results in diameters lower than the dimensions of the human scala tympani [31]. This indicates that the usage of a swelling actuator does not bear the danger of damaging the basilar membrane by shear force through swelling. Additionally, the implant will not completely fill the space in the scala tympani and therefore will not induce the risk of displacing the remaining perilymph by filling space, which could affect homeostasis.

### 3.3. Biocompatibility Tests

The tested composite samples displayed an open-pore and flexible surface. In the first in vitro investigations, cells were reluctant to grow on the surface, and microscopic evaluation was complicated due to cells growing in different dimensions. Furthermore, the absorption of the cell culture media by the samples quickly left no conditions for cell proliferation. Since the aim of the tests was to evaluate the materials’ biocompatibility and not the cell growth on the material surface, a WST test was completed using cell culture media conditioned with the composites.

**Figure 12:**
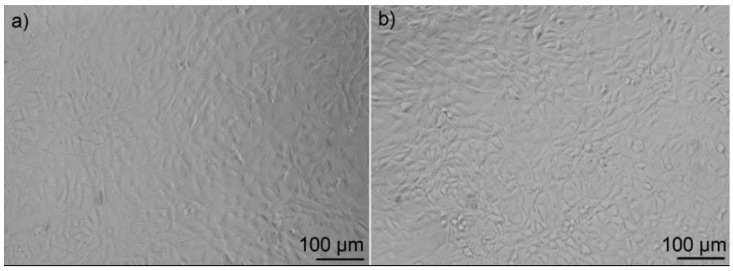
Light microscopy images of a layer of NIH-3Te fibroblast cells grown in well plates for evaluation of cell morphology and proliferation: (**a**) cells in media conditioned with Nusil Med 4850-PAMAA composite samples and (**b**) the negative control. Cells in both wells showed normal morphology and proliferated as expected.

Figure 12 shows light microscopy images of the cell morphology and proliferation in a well with conditioned media (Figure 12a) and the negative control (Figure 12b). Due to good cell proliferation, a cell layer of 100% confluence was developed.

**Figure 13:**
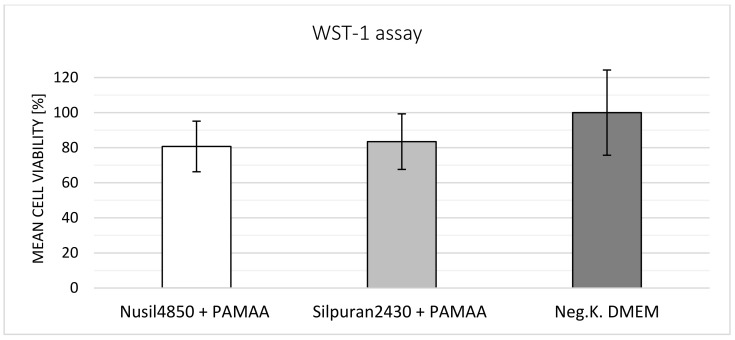
WST-1 biocompatibility results of Silpuran 2430-PAMAA and Nusil 4850-PAMAA samples repeated four times with *n* = 6 samples, mean ± standard deviation, and negative control. Mean cell viability for all repetitions corresponding to each material combination was calculated. All composites, irrespective of particle size and percentage of the hydrogel phase, yielded the same result of over 70% cell viability.

The cell viability results of each test repetition with *n* = 6 samples were evaluated. Results of the third repetition were considerably lower, with 51 ± 5.1% for Silpuran 2430 + PAMAA and 58.2 ± 7.6% for Nusil Med 4850 + PAMAA when compared to the general results obtained. This could have been due to unintentional experimental faults. Except for the situation mentioned above, Silpuran 2430-PAMAA samples showed cell viability higher than 70%. In Figure 13, a summary of WST-1 tests with each material combination and the negative control is plotted. While cells cultured in Nusil 4850-PAMAA-conditioned medium exhibited a mean cell viability of about 80%, the Silpuran composite showed a slightly higher value of 83%.

These high cell viabilities of more than 70% are generally declared to be non-toxic sample reactions and are positive for a first biocompatibility evaluation. However, further testing of possible interaction reactions of silicone rubber with PAMAA has to be performed according to ISO 10993-5. In addition, long-term biocompatibility tests also have to be performed in future experiments.

To our knowledge, reports in the literature on the biocompatibility of pure PAMAA do not exist. However, nanocomposite hydrogels based on PAMAA have been investigated as biomaterials for tissue engineering. When implanted subcutaneously in mice, these nanocomposite hydrogels showed good biocompatibility with the absence of an immune response [32].

## 4. Conclusions and Outlook

A curved unwired cochlear implant shaft based on a PAMAA hydrogel–PDMS rubber composite as a swelling actuator with bending radii close to the typical apical geometry of human cochlea was successfully manufactured. Even though the crosslinked PAMAA hydrogel is solely characterized by a maximum water uptake of 400%, its mixture with PDMS (with an initial hydrogel content ranging from 20 to 30 wt%) led to swelling ratios of up to 375% (Silpuran 2430) and up to 190% (Nusil Med 4850). However, in a swollen state, the hydrogel–PDMS composite was still able to exert the necessary bending forces on a PDMS rubber substrate to which the composite was deposited and adhered. The internal interfaces of swelling and stretched materials withstand these forces well and steadily. In addition, the only slight volume increase suggests no danger of damaging the basilar membrane by too much swelling nor misbalancing the homeostasis by reducing the free space in the scala tympani. In the final design, CI arrays with the possible variants of internal connecting wires must still be tested. Here, there is a chance that the fine wires could minimize the elongation of the pure silicone rubber, which, according to our estimations, could further improve the curvature properties of the electrode shafts. Additional potential for optimization lies in the use of higher particle concentrations with smaller particle size fractions if these become able to be processed. However, further challenges of biocompatibility have to be considered. Modified hydrogels with similar swelling capacities have to be developed as well as methods to process hydrogel fractions with a very low particle size (in our case, <28 µm) to avoid agglomeration as an undesirable phenomenon.

## Figures and Tables

**Table 1:** Material properties of Silpuran 2430 and Nusil Med 4850 [17,18].

	Silpuran 2430	Nusil Med 4850
Curing	10 min at 135 °C	5 min at 150 °C
Biocompatibility	Restricted (<28 days)	Unrestricted
Polymerization	Room temperature polymerized (RTV 2)	Liquid Silicone Rubber (LSR)
Hardness Shore	20	50
Tensile strength	6 MPa	10.17 MPa
Elongation at rupture	540%	675%

1 ISO 888; 2 According to ISO 10993-5 no cytotoxicity.

**Table 2:** List of manufactured samples with designs according to Figure 2. The investigated silicone rubbers are given for each design, as well as the PAMAA particle size and the respective weight percentage used in the compounds.

PAMAA Particle Size	Layer Height	Material Combinations	
Samples of design A		Silpuran 2430 + PAMAA|	Nusil Med 4850 + PAMAA
<20 µm		20 wt%, 25 wt%, 30 wt% *n* = 6 samples each	
20–50 µm			
50–100 µm			
Samples of design B		Nusil Med 4850 + PAMAA	
20–50 µm		20 wt%|*n* = 6 samples	
50–100 µm		20 wt%, 25 wt%, 30 wt%|*n* = 6 samples each	
Samples of design C		Nusil Med 4850 + PAMAA	
20–50 µm	Basal 0.65 mm Apical 0.30 mm	20 wt%|*n* = 3 samples	

**Table 3:** Laser diffraction results of hydrogel particle size distribution of the grain size mass fractions <20 µm, 20–50 µm, and 50–100 µm. Three averaged median values (*n* = 3) for each size distribution are shown.

	50–100 (µm)	20–50 (µm)	0–20 (µm)
Median (x_50_)	77.39	48.00	28.34
Standard deviation	6.38	1.01	1.26

**Table 4:** Curvature radii determined for samples of designs B and C.

Hydrogel Content (wt%)	Grain-Size Fraction (µm)	Individual Radii for the Samples Prepared (S1–S6) (mm)			Mean Radius (mm)
Design B					
20	48	S1—17.4	S2—7.7	S3—7.5	10.9
		S4—8.7	S5—9.8	S6—14.4	
20	77	S1—15.9	S2—12.0	S3—12.6	12.2
		S4—5.6	S5—12.3	S6—14.8	
25	77	S1—14.4	S2—21.2	S3—16.2	16.6
		S4—16.5	S5—16.2	S6—15.2	
30	77	S1—13.7	S2—17.5	S3—13.3	14.8
		S4—14.0	S5—11.4	S6—18.8	
Design C					
20	48	S1		basal section—3.9	3.6
				middle section—3.9	
				apical section—3.1	
20	48	S2		basal section—2.5	1.9
				middle section—1.25	
				apical section—1.87	

## Data Availability

Not applicable.

## Appendix A

### Appendix A.1. Sample Preparation

The grinding process was started with two grinding balls with a 15 mm diameter for 30 min with a frequency of 30 s^−1^. Subsequently, the material was ground with 30 grinding balls with a 5 mm diameter at a frequency of 30 s^−1^ for 50 min. After grinding, the powder was sieved using a vibratory screening machine (Analysette 3 Pro Fritsch, Idar-Oberstein, Germany) with an amplitude of 1 mm and a sieving duration of 60 min. Sieve mesh sizes of 100 µm, 50 µm, and 20 µm were used. Due to the rapid agglomeration of the hydrogel particles, the sieves became clogged often and had to be cleaned with purified water in an ultrasonic bath. All powder with the desired grain-size fraction was stored. The rest was ground again following the described procedure.

### Appendix A.2. Biocompatibility Tests

By determining the metabolic activity of native murine NIH-3T3 fibroblasts, the samples were evaluated in line with ISO 10993-5. Due to the high water absorption of the hydrogel, tests were performed with conditioned media instead of direct sample contact. For each material (Silpuran 2430-PAMAA and Nusil Med4850-PAMAA), six samples consisting of 0.8 mm^2^ silicone rubber–hydrogel composite were tested. The samples were autoclaved at 121 °C for 20 min in a 100 mL wide-necked Schott bottle. Under sterile conditions, 20 mL of cell culture medium consisting of Dulbecco’s modified Eagle’s medium (DMEM, Biochrom, Berlin, Germany) with 10% fetal calf serum (FCS, Biochrom, Berlin, Germany) and 0.5% L-Glutamin (Biochrom, Berlin, Germany) was added to the bottle and incubated with the sample under cell culture conditions (37 °C/5% CO_2_) for seven days in a slightly open bottle.

The conditioned cell culture medium was then aliquoted and frozen at −20 °C. For the WST-1-Assay, 10,000 fibroblasts per well were cultivated for 48 h at 37 °C and 5% CO_2_ in the conditioned cell culture media (100 µL per well), the negative control (unconditioned cell culture medium, proliferation under normal conditions), and the positive control (DMSO, induces cell death). Then, 10 µL of WST-1 solution was added to each well and incubated again for 30 min (37 °C, 5% CO_2_). After the incubation period, the cleavage of the tetrazolium salt WST-1 to formazan was measured with a multi-well spectrophotometer (ELISA reader) and determined quantitatively. The measured absorbance correlated directly with the number of viable cells, calculated by the mean optical density of a certain sample and the negative control wells, which were set at 100% cell viability.
